# Specific Nutrients Mediate the Association of Food Insecurity and Sleep Regularity Index (SRI) in U.S. Adults: NHANES 2011–2014

**DOI:** 10.3390/nu17020340

**Published:** 2025-01-18

**Authors:** Samuel Myron Degenhard, Nicole Farmer, Li Yang, Jennifer J. Barb, Katherine A. Maki, Gwenyth R. Wallen

**Affiliations:** Translational Biobehavioral and Health Disparities Branch, Clinical Center, National Institutes of Health, Bethesda, MD 20892, USA; sam.degenhard@nih.gov (S.M.D.);

**Keywords:** food insecurity, sleep regularity index, nutrition security, food assistance programs, SNAP, macronutrients, micronutrients, fatty acids, health disparity, NHANES

## Abstract

Background/Objectives: Food-insecure individuals are at risk for poor health outcomes, including substandard sleep health. A possible association of food insecurity with sleep regularity has not been explored, and factors contributing to the relationship between food insecurity and sleep are not well understood. This cross-sectional study explored the relationship between food insecurity and sleep regularity and identified specific nutrients that mediated the association. Methods: This study used dietary intake, interview, physical examination, actigraphy, and laboratory data from NHANES 2011–2014 to assess the possible correlation between food insecurity and sleep in a sample of U.S. adults (*n* = 6730). Mediation analysis was conducted to determine specific serum biomarkers and intake of nutrients that indirectly contributed to the relationship. Results: Food insecurity was negatively correlated with sleep regularity. Dietary intake of fiber; vitamins A, B1, B2, C, E, and K; β-carotene; zinc; copper; and potassium and serum concentrations of palmitoleic acid had significant indirect effects on this association. The low/marginal food security group under-consumed vitamin K, and the very low food security group under-consumed vitamin K and zinc. Among food-insecure, income-eligible adults, those who received benefits from food assistance programs consumed significantly less fiber and β-carotene and exhibited significantly higher serum concentrations of palmitoleic acid than non-participants. Conclusions: Food insecurity predicted sleep regularity, and this relationship was mediated by dietary intake and serum concentrations of specific nutrients, underscoring the role of nutrition security when evaluating potential health impacts for adults experiencing food insecurity.

## 1. Introduction

Food insecurity (FI) is a socioeconomic condition of limited access to adequate quantities of nutritious food [1]. FI represents a severe global health concern, and it is estimated that FI impacted 2.33 billion people around the world in 2023 [2]. FI is associated with poor health outcomes, including a higher risk of hypertension, hyperlipidemia, diabetes, obesity, cardiovascular disease, and all-cause mortality [3,4,5,6,7].

Additional factors such as diet quality and physical activity may moderate the relationship between FI and health [8,9]. Less clear is the role FI plays in sleep quality, a critical yet often-overlooked health outcome [10,11]. Previous studies have linked FI to trouble falling asleep, either shorter or longer sleep duration, and worse sleep quality [12,13,14,15,16].

Much of the literature focuses on studying sleep duration and quality. To date, no previous study has assessed the relationship between FI and sleep regularity, a measure of the consistency of sleep patterns based on innate circadian rhythms [17]. Sleep regularity index (SRI) is a validated metric calculated from objective actigraphy data and used to assess sleep–waking timing. Inconsistent sleep–wake timing can disrupt circadian rhythms, possibly leading to obesity, compromised immune function, diabetes, cardiovascular disease, and psychiatric disorders [18,19,20]. Thus, sleep regularity is highly interconnected with wellness and chronic disease, and it is a stronger predictor of mortality than sleep duration [21,22,23,24,25,26].

Additionally, previous studies have strongly suggested that diet composition and nutritional biomarkers could be important factors in predicting sleep behavior. For instance, a higher energy distribution from carbohydrate and fat and a lower intake of protein and dietary fiber have been linked to poor sleep quality and sleep–wake regularity [27,28,29,30]. Previous work has identified micronutrient status, including the intake of vitamins A, B12, C, D, E, and K; magnesium; niacin; calcium; iron; copper; and zinc, as predictors of sleep quality [31,32,33]. It has been demonstrated that serum folate, zinc, iron, and fatty acid concentrations, specifically palmitic, palmitoleic, oleic, and linoleic acids, were negatively correlated with sleep disturbances and poor sleep quality [34,35,36]. However, the relationship of nutritional status and sleep has not been studied in food-insecure populations.

Like sleep, FI is related to dietary factors, such as a lower intake of protein; fat; dietary fiber; vitamins A, B6, and B12; folate; magnesium; phosphorus; niacin; copper; riboflavin; and zinc and higher carbohydrate consumption [6,37,38,39,40,41]. If, as the literature suggests, nutrition is implicated in sleep behaviors, then it is reasonable that the variety of nutritional deficiencies to which food-insecure populations are especially vulnerable might contribute to the reported relationship between FI and sleep.

Similarly, food assistance programs (FAPs) such as the Supplemental Nutrition Assistance Program (SNAP) or food stamps might exacerbate dietary shortcomings related to FI. For example, it has been demonstrated that SNAP participants have worse diet quality than income-eligible non-participants [42]. Therefore, FAP participation could impact intake or serum concentrations of nutrients related to FI and sleep, possibly modulating the relationship of diet and sleep in food-insecure populations.

Yet, the role of nutrition in the link between FI and sleep quality is not fully understood. Therefore, using data from the National Health and Nutrition Examination Survey (NHANES) 2011–2014, the present study sought to evaluate the relationship between FI and sleep regularity and to determine whether this relationship was mediated by intake and the serum concentrations of nutrients. A secondary aim was to evaluate the impact of FAPs on the consumption or serum concentrations of the identified nutrients among food-insecure adults. It was predicted that food-secure individuals would exhibit higher sleep regularity than their food-insecure counterparts and that nutritional deficiencies relevant to FI would mediate this association. It was also hypothesized that, among food-insecure, income-eligible individuals, FAP participants would consume less or exhibit lower serum concentrations of the mediating nutrients compared to non-participants.

## 2. Materials and Methods

### 2.1. Study Sample

**Figure A1:**
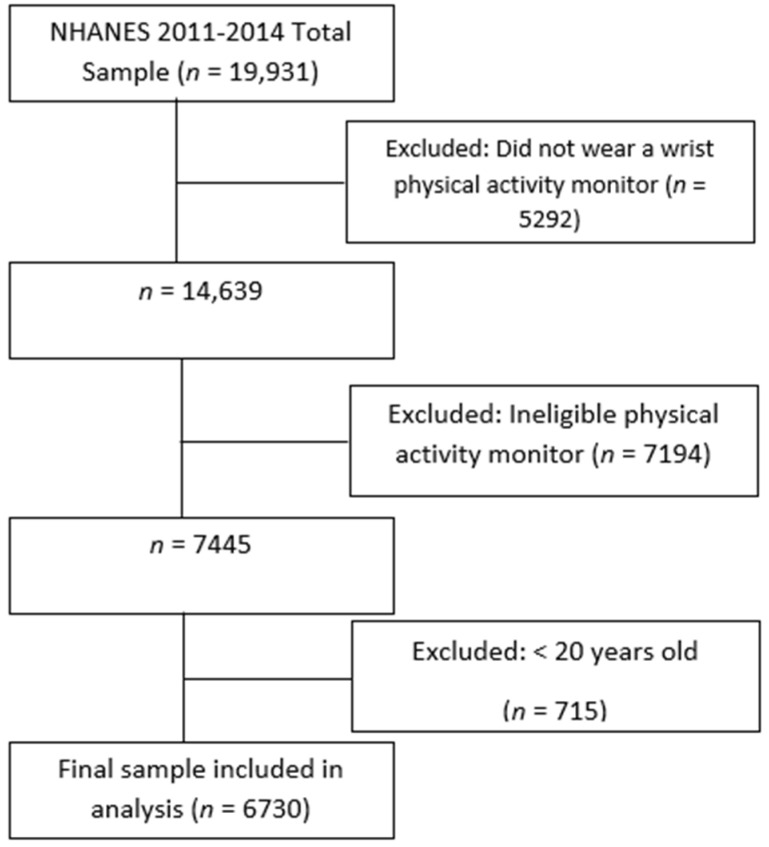
Filtering of participants for final study sample.

NHANES is a cross-sectional, nationally representative health and nutrition survey administered by the National Center for Health Statistics (NCHS) at the Centers for Disease Control and Prevention [43]. It includes interviews, physical examinations, and laboratory tests of noninstitutionalized U.S. civilians. The study population was taken from the NHANES 2011–2012 and 2013–2014 surveys, as these cycles were the most recent to collect actigraphy data necessary to determine SRI scores. The NHANES sample was selected using a complex, multistage design that oversampled low-income people, non-Hispanic Asian and Black people, and those over the age of 80 years. The sample weights of the 2011–2012 and 2013–2014 release cycles were combined to create new four-year sample weights for interview, physical examination, and laboratory test sample persons [44]. The sample weights provided estimates that were representative of the U.S. population. Of the 19,931 unweighted participants, only those over the age of 20 years with available actigraphy and dietary data were included (see Figure A1). The final study sample included 6730 adults.

### 2.2. Food Security Status

NHANES 2011–2014 included the U.S. Food Security Survey Module, a validated 18-item questionnaire that assesses household food insecurity experienced in the last 12 months [45]. The module consisted of a series of questions that participants answered affirmatively or negatively. Only the ten household adult items in the scale were considered, as the remaining eight were specific to children. Accordingly, participants were considered fully food-secure if they did not answer affirmatively to any of the items. Participants were classified as marginal, low, or very low in terms of food security if 1–2 items, 3–5 items, or 6–10 items were affirmative, respectively. Marginal and low food security groups were combined because of their small proportion in the total sample and lack of significant between-group differences of SRI scores.

### 2.3. Actigraphy Data and Sleep Regularity Index Calculation

Actigraphy data were collected using a physical activity monitor (ActiGraph model GT3X+, manufactured by ActiGraph, Pensacola, FL, USA) that participants wore on their wrist for seven days. Wrist actigraphy provides highly accurate and sensitive measures of sleep and is particularly useful for evaluating circadian rhythms [46,47]. The monitor recorded continuous accelerometer and ambient light information. These actigraphy data provided objective estimates of sleep–wake patterns and the timing of participants based on horizontal axis orientation and light exposure. Actigraphy data were used to determine the proportions of wakefulness and sleep during the day and night. Subsequently, the sleep regularity index (SRI) was calculated to represent the night-to-night shifts in sleep onset and offset. The formula for the SRI calculated in these data was first introduced by Lundsford-Avery et al. [48].

The SRI developed by Phillips et al. [49] is described as the “percentage probability of an individual being in the same state (asleep vs. awake) at the same two time-points 24 h apart”. Actigraphy data included in the SRI calculation required that a participant had data for seven days. Participants that did not wear the physical activity monitor for more than two hours in a single day were excluded. Epochs from the activity monitor were measured as one-minute intervals (1440 epochs for each 24 h period). The percentage of day/night wake and day/night sleep was calculated using the indicator ‘wake’ or ‘sleep’ from the actigraphy files. The percentage was calculated as the total number of sleep or wake epochs divided by the total epochs for that day or night interval. Day intervals were collected between 9:31 a.m. and 9:30 p.m. and night intervals from 9:31 p.m. to 9:30 a.m.

### 2.4. Nutrient Dietary Intakes

Nutrient intakes, including total daily caloric intake, were obtained from 24 h dietary recalls [50]. NHANES includes data from two dietary recalls; however, nutrient data from only the first recall were analyzed. The first recall was conducted in person during the medical examination visit on the first day that actigraphy data were collected. Each interview utilized a validated multi-pass method for dietary intake evaluation. The selection of available nutrients from the dietary recall interview database to include in this analysis was informed by existing literature on sleep behaviors and sleep quality (see Table A1 for references).

### 2.5. Serum Nutrient Levels

Blood was drawn from participants at the medical examination visit to determine serum concentrations of nutrients. Participants were requested to fast for nine hours prior to blood collection.

### 2.6. Demographic Variables

Sociodemographic variables were age, gender (male, female), race and ethnicity (non-Hispanic White, non-Hispanic Black, Mexican-American/Other Hispanic, non-Hispanic Asian American/Other), marital status (married/living with partner, unmarried), education level (high education, low education), and employment status (employed, unemployed). Participants were considered employed if they reported working a job or business in the week prior to data collection. For race and ethnicity, Mexican-American and Other Hispanic, as well as non-Hispanic Asian American and Other were combined after determining no differences between groups in SRI.

### 2.7. Physical and Mental Health Variables

Health- and diet-related factors, including BMI (kg/m^2^), depressive symptoms (no symptoms, any symptoms), diabetes (diabetic, not diabetic), blood pressure (high blood pressure, normal blood pressure), and total daily calorie intake, were controlled for. Depression was assessed using the Patient Health Questionnaire-9 (PHQ-9), a validated nine-item measure of depressive symptoms developed by Kroenke et al. [51]. Participants were considered to exhibit depressive symptoms if their PHQ-9 score was above four. Additionally, diabetes and blood pressure were self-reported based on the following respective survey items: “Have you ever been told by a doctor or other health professional that you have diabetes or sugar diabetes?” and “Have you ever been told by a doctor or other health professional you have high blood pressure?”.

### 2.8. Food Assistance Program Participation

Receipt of food stamp assistance was determined by a single question assessing if participants had ever received food stamp benefits.

### 2.9. Statistical Analysis

Complex sampling procedures with combined sample weights were used to account for the NHANES complex sample design. Weighted means, proportions, and standard errors were used to describe the overall sample and the FI groups. Complex samples Pearson χ^2^ tests and complex samples general linear models were used to assess possible differences in categorical and continuous variables between FI groups, respectively. Unadjusted and adjusted complex samples general linear models were constructed to determine the potential association between FI status and SRI score. The literature was searched in an unsystematic fashion to identify potential nutrients or biomarkers implicated in sleep behavior. In total, 41 nutrients that were possibly related to sleep were included in the analysis.

Nutrient intake and serum biomarker variables were natural log transformed to reduce skewness. Additional complex samples general linear models were used to determine whether each nutrient was significantly associated with SRI scores and FI status. Variables that were related to both FI status and SRI score were then tested for potential mediating effects in the relationship between FI and SRI using the PROCESS Version 4 SPSS macro [52]. Potential differences between FI groups of intake or serum concentrations of the mediating nutrients were assessed. Also, differences in intake and serum concentrations of the mediating nutrients among food-insecure, income-eligible FAP participants and non-participants were also examined. Individuals were considered food insecure if they were part of the marginal, low, or very low food-secure groups, and household income eligibility for food assistance was set at < 130% of the U.S. Department of Health and Human Services (HHS) monthly poverty index. The HHS poverty index is specific to the year, U.S. state, and family size of each participant [53]. *p*-values under 0.05 were considered statistically significant.

## 3. Results

Our sample included 6730 adults aged 20 to 88 years, 30.3% of which were food-insecure, 52% were female, and 66.2% were non-Hispanic White (see Table 1). Marginal/low and very low food-secure participants were less likely to be married, have a job, have earned a 4-year college degree, and were more likely to have participated in FAPs and identify as a person of color than fully food-secure counterparts. Moreover, the marginal/low and very low food security groups had significantly higher proportions of participants reporting to be obese, experience depressive symptoms, and sleep less than six hours per day and had higher average BMI than the full food security group.

Across the sample, the mean SRI score was 61.3 (±0.37), and the average proportions of nighttime wake, nighttime sleep, day wake, and day sleep were 40.5% (±0.19), 59.5% (±0.19), 90.6% (±0.18), 9.91% (±0.18), respectively. FI status was significantly associated with SRI score, regardless of age, gender, race/ethnicity, marital status, employment status, FAP participation, diabetes, depressive symptoms, blood pressure, BMI, and daily caloric intake (Table 2). In fully adjusted models, estimated marginal means indicated that food-secure individuals had 9.26% higher SRI scores than their very low food-secure counterparts (*p* < 0.001) and no difference with the marginal/low food security group (*p* = 0.08).

Of the 41 nutrients examined based on the existing literature, 31 had significant associations with SRI score in unadjusted models (Table A1). FI exhibited a significant relationship with intake or serum concentrations of 23 nutrients (Table A2), leaving 16 nutrients with significant relationships with both the SRI score and FI status. After testing for mediating effects in models controlling for possible confounding variables, 12 of the 18 nutrients were shown to have significant indirect effects on the relationship between FI and SRI (Table 3). Palmitoleic acid displayed a significant negative correlation with SRI, while fiber; vitamins A, B1, B2, C, E, and K; β-carotene; zinc; copper; and potassium were significantly positively associated with SRI (see Table A1).

Comparisons of sample mean intakes or serum concentrations of nutrients to dietary guidelines and laboratory reference ranges indicated that all groups did not consume adequate fiber and vitamins A and C (Table 4). Additionally, marginal/low food-secure individuals under-consumed vitamin K, and very low food-secure participants under-consumed vitamin K and zinc. Overall, and within each food security status group, participants consumed sufficient amounts or exhibited normal serum concentrations of the remaining mediating nutrients. Among food-insecure, income-eligible participants (*n* = 2119), those that had ever received benefits from FAPs consumed significantly less fiber and β-carotene and exhibited significantly higher serum concentrations of palmitoleic acid than counterparts that had never received benefits (Table 5).

## 4. Discussion

This analysis of NHANES 2011–2014 showed that FI is negatively correlated with objectively measured sleep regularity and that specific nutrients that differ by FI status may mediate this association. The results of the present study add to the growing body of evidence substantiating the link between FI and poor sleep behavior [14]. Additionally, this study underscores the importance of the intake of specific nutrients that may contribute to this association and should be considered when evaluating the potential health impact of FI in adults.

Although the biological and psychosocial mechanisms behind the FI–sleep relationship have not been fully elucidated, one existing hypothesis posits that mental health issues, which are more prevalent in food-insecure populations, are at least partially responsible for associated sleeping problems [14]. Our study supports an additional explanation for the association between FI and sleep behavior, one that indicates that diet-related factors could indirectly lead to unhealthy sleep behavior in food-insecure individuals. Specifically, our results emphasize the importance of stable access to food of adequate nutritional value, a concept known as nutrition security [56].

All groups under-consumed fiber and vitamins A and C, the marginal/low food security group did not meet daily intake requirements of vitamin K, and the very low food security group under-consumed vitamin K and zinc. This suggests that those experiencing FI are more likely to under-consume these target nutrients, which could possibly lead to the observed sleep problems.

Similarly, it was observed that among those who were food-insecure and income-eligible for FAPs, benefits receivers consumed significantly less fiber and β-carotene and had higher serum concentrations of palmitoleic acid than non-receivers. This finding suggests that FAPs could possibly modulate how FI impacts sleep regularity by altering the foods, and, in turn, nutrients, that individuals consume. Our results are also consistent with previous work demonstrating diet quality disparities between FAP participants and non-participants [42], mirroring a broader public health phenomenon of FAP participation and adverse health behaviors [57,58]. In line with publications illustrating the role of behavioral economics on influencing food choice among FAP recipients, our results indicate a potential need for education and behavioral interventions targeting diet quality, specifically the intake of nutrients that could improve sleep outcomes to further promote overall health. However, other factors need to be considered when interpreting our results, such as the prevalence of FI among FAP recipients, interruptions in assistance to recipients, and varying monetary amounts of assistance provided to recipients based on geographic location, family size, and other factors.

In contrast to Irmisch and colleagues, this study showed that the serum concentration of palmitoleic acid was negatively correlated with sleep regularity. Irmisch and colleagues attributed their observed association of palmitoleic acid and fewer sleep disturbances to the existence of these fatty acids as a precursor to the sleep-inducing fatty amide, oleamide [35]. However, emerging research utilizing fly models found that elevated palmitic acid intake, a precursor of palmitoleic acid, disrupts sleep patterns and alters gut microbiota composition, leading to intestinal inflammation and the dysregulation of neurotransmitter production [59]. Although more research, particularly with humans, is warranted, FI has been associated with a high-carbohydrate diet, which has been demonstrated to promote palmitic acid synthesis via endogenous de novo lipogenesis, potentially disrupting the gut microbiome and sleep [54,55,60].

Additional biological mechanisms may further clarify the findings of the present study. For example, vitamins B1 and B2 were found to be significant mediators of the FI–SRI relationship, further implicating these nutrients in sleep behavior [31,32,33]. Earlier work has established the role of these vitamins in the production of sleep-related neurotransmitters including dopamine, serotonin, and acetylcholine, which may explain their relationship with sleep regularity [61].

Additionally, β-carotene and vitamins A, E, and K exhibit antioxidative properties that have the potential to reduce stress-related inflammation and are correlated with sleep quality [62]. The identification of potassium intake as a significant mediator of the association between FI and sleep regularity also supports previous studies linking this essential mineral to a tighter coupling of sleep–wake timing [63,64]. Finally, dietary fiber was a significant mediator, paralleling existing work showing that fiber intake is associated with favorable sleep outcomes [29]. Specifically, butyrate, a product of microbial fermentation of fiber in the lower intestinal tract, has been shown to upregulate the expression of circadian clock genes [65].

### Limitations and Future Directions

The present study included several methodological limitations that should be considered. The cross-sectional nature of our analysis prevented the establishment of potential causal relationships. Similarly, the limitations of NHANES demographic and health data meant confounding factors, such as smoking status, controlled substance usage, geographic location, and family composition, could not be effectively controlled. In addition, this study only included nutrients for analysis that were identified in the literature to be related to sleep behavior. Therefore, it is possible that other potential mediating nutrients were not tested in this study.

Self-reported dietary recall is subject to recall and social desirability biases and is prone to inaccuracies such as the underreporting of energy intake [66]. Similarly, food sources of the mediating nutrients were not assessed to link nutrients to the types of food consumed. Next, actigraphy and diet recall data provided only snapshots of participants’ sleep and dietary behaviors. Despite the FI classification reflecting an individual’s food access over a period of 12 months, FI status is a lived experience and can be dynamic over a 12-month period. Responses to the FI module may not be congruent with the eating habits of the single day or week in which the diet recall was conducted or the actigraphy data were collected. Moreover, although physical activity monitors have been demonstrated to accurately measure sleep [46], subjective sleep diaries were not available to corroborate the sleep estimates generated from actigraphy data.

The results of this study may not reflect the current realities of FI, nutrition, and FAPs in the United States, and the role of FAPs in the consumption and serum concentrations of mediating nutrients should be reevaluated using more recent data. For example, several changes to food stamps/SNAP legislation have occurred since the 2011–2014 NHANES cycles, including an expansion of eligibility, increased benefit amounts, and the option for online grocery delivery and pickup [67].

Future research is needed to comprehensively evaluate whether the daily intake requirements of nutrients related to sleep regularity are satisfied among those experiencing FI, emphasizing the role of food environments and FAPs in diet quality and health. Further, the role of smoking and controlled substance use, factors known to relate to sleep outcomes, should also be explored in future studies. To provide practical dietary guidance, future research analyzing the food sources of the mediating nutrients is warranted. Also, causal relationships between specific nutrients and sleep regularity should be established by utilizing longitudinal cohorts and further exploring the potential biological mechanisms underlying these associations and mediating effects.

## 5. Conclusions

In summary, the current study established a novel link between FI and sleep regularity and identified that the intake of fiber; vitamins A, B1, B2, C, E, K; β-carotene; zinc; copper; and potassium and serum concentration of palmitoleic acid partially mediated this relationship. Our results add to the growing body of evidence pointing to the detrimental impacts of FI on various dimensions of health, including sleep. This study suggests that FI status does not tell the entire story but, rather, diet and serum biomarkers commonly associated with a lack of access to food may play a role in predicting sleep behavior. Specifically, our results illuminate potential food-insecurity-related circadian rhythm disruptions and indicate that food assistance programs exacerbate diet quality disparities that may be implicated in sleep. As such, nutrition security status should be an important consideration when evaluating individual- and population-level health in clinical and epidemiological contexts and designing FAPs to address FI and diet more effectively. These results may serve to advance practice in promoting healthy behaviors by designing interventions that simultaneously address changes in multiple health behaviors, such as nutrition, in the context of FI and sleep.

## Figures and Tables

**Table 1:** Participant characteristics by food insecurity level.

	Total	Full Security	Marginal/Low Security	Very Low Security
	*n* = 6730	*n* = 4689 (69.7%)	*n* = 1509 (22.4%)	*n* = 532 (7.9%)
Age, y	47.5 (±0.45)	49.1 (±0.47)	42.6 (±0.56) ^1^	41.2 (±0.72) ^1^
Gender, %				
Male	48.0 (±0.50)	48.4 (±0.50)	46.2 (±1.2)	48.7 (±3.2)
Female	52.0 (±0.50)	51.6 (±0.50)	53.8 (±1.2)	51.3 (±3.2)
Employed, %	61.3 (±1.0)	62.9 (±1.1)	57.9 (±1.8) ^1^	52.6 (±1.9) ^1^
Married, %	61.8 (±1.2)	65.1 (±1.2)	53.4 (±1.9) ^1^	46.5 (±3.1) ^1^
Ever Received Food Assistance Benefits, %	24.2 (±1.6)	14.5 (±1.3)	48.5 (±2.4) ^1^	68.0 (±3.6) ^1^
Poverty Level, %				
<130%	27.0 (±1.4)	17.4 (±1.2)	53.4 (±2.6) ^1^	64.3 (±3.1) ^1^
130–185%	12.1 (±0.50)	11.0 (±0.60)	14.6 (±1.2) ^1^	17.8 (±2.8) ^1^
>185%	60.9 (±0.1.7)	71.6 (±1.5)	32.0 (±2.6) ^1^	17.9 (±2.2) ^1^
Race, %				
Non-Hispanic White	66.2 (±2.5)	71.6 (±2.2)	47.2 (±3.7) ^1^	54.8 (±4.0) ^1^
Non-Hispanic Black	11.5 (±1.4)	9.30 (±1.1)	18.5 (±2.2) ^1^	17.4 (±2.6) ^1^
Non-Hispanic Asian/Other	7.90 (±0.60)	8.20 (±0.60)	7.0 (±1.2) ^1^	7.30 (±1.3) ^1^
Mexican-American/Other Hispanic	14.5 (±0.1.7)	10.9 (±1.5)	27.3 (±3.1) ^1^	20.6 (±3.1) ^1^
Education, %				
Some College or Lower	69.4 (±1.6)	62.9 (±1.8)	87.9 (±1.2) ^1^	93.7 (±1.6) ^1^
College Degree or Higher	30.6 (±1.6)	37.1 (±1.8)	12.1 (±1.2) ^1^	6.3 (±1.6) ^1^
BMI	28.9 (±0.13)	28.6 (±0.16)	29.8 (±0.20) ^1^	30.5 (±0.41) ^1^
Obese, %	36.5 (±0.80)	34.3 (±1.0)	42.2 (±1.3) ^1^	45.1 (±2.1) ^1^
Hypertensive, %	33.6 (±0.90)	33.6 (±1.0)	32.6 (±1.4)	35.2 (±2.2)
Diabetic, %	9.70 (±0.40)	9.20 (±0.50)	10.7 (±0.70)	12.1 (±0.1.6)
Depressive Symptoms, %	66.1 (±0.60)	63.3 (±0.70)	73.0 (±1.1) ^1^	79.4 (±1.8) ^1^
Household size	3.2 (±0.02)	3.0 (±0.02)	3.7 (±0.07)	3.4 (±0.08)
Kcal per day	2170 (±12)	2150 (±15)	2160 (±29)	2280 (±90)
<6 h of Sleep per Day, %	35.9 (±0.80)	33.5 (±0.80)	41.1 (±1.6) ^1^	49.7 (±1.8) ^1^
SRI Score	61.3 (±0.37)	62.5 (±0.40)	58.7 (±0.55) ^1^	52.8 (±1.0) ^1^

^1^ Significant difference from full food security group (*p* < 0.05).

**Table 2:** Effect of food insecurity status on SRI score.

Model	*F*(2, 31)	*R* ^2^	*p*	Marginal/Low Contrast Estimate	Very Low Contrast Estimate
Unadjusted	52.2	0.029	<0.001	−3.80 (±0.66) ^1^	−9.70 (±1.1) ^1^
Demographic ^2^ Covariates	18.4	0.138	<0.001	−1.69 (±0.61) ^1^	−6.08 (±1.0) ^1^
Demographic + Health ^3^ Covariates	11.1	0.171	<0.001	−1.28 (±0.70)	−5.40 (±1.2) ^1^

^1^ *p* < 0.05. ^2^ Age, gender, race/ethnicity, education level, employment status, FAP participation, and marital status. ^3^ Hypertension, diabetes, depressive symptoms, daily caloric intake, and BMI.

**Table 3:** Mediating effects of intakes or serum concentrations of nutrients in adjusted models ^1^.

	Marginal/Low Security			Very Low Security		
	β-Total [95% CI]	β-Direct [95% CI]	β-Indirect [95% CI]	β-Total [95% CI]	β-Direct [95% CI]	β-Indirect [95% CI]
Dietary Fiber	−1.34 [−2.3–−0.38]	−1.26 [−2.2–−0.31]	−0.078 [−0.16–−0.01] ^1^	−4.44 [−5.9–−3.0]	−4.40 [−5.8–−3.0]	−0.035 [−0.15–0.07]
Vitamin A	−1.34 [−2.3–−0.33]	−1.29 [−2.2–−0.33]	−0.050 [−0.12–−0.01] ^1^	−4.44 [−5.9–−3.0]	−4.33 [−5.8–−2.9]	−0.105 [−0.22–−0.02] ^1^
Vitamin E	−1.34 [−2.3–−0.38]	−1.26 [−2.2–−0.31]	−0.076 [−0.14–−0.02] ^1^	−4.44 [−5.9–−3.0]	−4.37 [−5.8–−2.9]	−0.070 [−0.17–−0.01] ^1^
Vitamin C	−1.34 [−2.3–−0.38]	−1.33 [−2.3–−0.38]	−0.004 [−0.05–0.04]	−4.44 [−5.9–−3.0]	−4.37 [−5.8–−2.9]	−0.074 [−0.18–−0.01] ^1^
Vitamin K	−1.34 [−2.3–−0.38]	−1.28 [−2.2–−0.33]	−0.058 [−0.12–0.01]	−4.44 [−5.9–−3.0]	−4.34 [−5.8–−2.9]	−0.100 [−0.21–−0.01] ^1^
Vitamin B1	−1.34 [−2.3–−0.38]	−1.27 [−2.2–−0.32]	−0.062 [−0.14–−0.01] ^1^	−4.44 [−5.9–−3.0]	−4.41 [−5.8–−3.0]	−0.027 [−0.13–0.06]
Vitamin B2	−1.34 [−2.3–−0.38]	−1.27 [−2.2–−0.32]	−0.065 [−0.13–−0.01] ^1^	−4.44 [−5.9–−3.0]	−4.36 [−5.8–−3.0]	−0.046 [−0.15–−0.03] ^1^
Vitamin B6	−1.33 [−2.3–−0.37]	−1.29 [−2.2–−0.34]	−0.039 [−0.10–0.01]	−4.45 [−5.9–−3.0]	−4.40 [−5.8–−2.9]	−0.077 [−0.19–0.01]
Potassium	−1.34 [−2.3–−0.38]	−1.28 [−2.2–−0.32]	−0.059 [−0.14–−0.01] ^1^	−4.44 [−5.9–−3.0]	−4.36 [−5.8–−2.9]	−0.010 [−0.01–0.01]
Beta-Carotene	−1.34 [−2.3–−0.38]	−1.33 [−2.2–−0.37]	−0.008 [−0.01–0.01]	−4.44 [−5.9–−3.0]	−4.35 [−5.8–−2.9]	−0.091 [−0.20–−0.01] ^1^
Choline	−1.33 [−2.3–−0.37]	−1.32 [−2.2–−0.32]	−0.008 [−0.04–0.01]	−4.45 [−5.9–−3.0]	−4.41 [−5.8–−3.0]	−0.043 [−0.13–0.02]
Zinc	−1.34 [−2.3–−0.38]	−1.27 [−2.2–−0.32]	−0.070 [−0.15–−0.01] ^1^	−4.45 [−5.9–−3.0]	−4.36 [−5.8–−2.9]	−0.094 [−0.21–−0.01] ^1^
Copper	−1.33 [−2.3–−0.37]	−1.28 [−2.2–−0.32]	−0.051 [−0.12–0.01]	−4.45 [−5.9–−3.0]	−4.31 [−5.7–−2.9]	−0.138 [−0.27–−0.04] ^1^
Total Folate	−1.33 [−2.3–−0.37]	−1.28 [−2.2–−0.32]	−0.053 [−0.13–0.01]	−4.45 [−5.9–−3.0]	−4.39 [−5.8–−3.0]	−0.061 [−0.18–0.04]
Serum Palmitoleic Acid	−2.20 [−3.6–−0.82]	−1.97 [−3.4–−0.59]	−0.232 [−0.42–−0.10] ^1^	−5.19 [−7.4–−3.0]	−4.94 [−7.1–−2.8]	−0.250 [−0.52–−0.05] ^1^
Serum Total Folate	−1.44 [−2.4–−0.47]	−1.38 [−2.3–−0.41]	−0.064 [−0.15–0.02]	−4.07 [−5.5–−2.6]	−3.95 [−5.4–−2.5]	−0.124 [−0.27–0.01]

Reference group: Full food security. Note. “Total”, “Direct”, and “Indirect” refer to the total, direct, and indirect effects respectively of each nutrient mediation model that controlled for age, gender, race/ethnicity, employment status, FAP participation, marital status, hypertension, diabetes, depressive symptoms, daily caloric intake, and BMI. ^1^ Significant indirect effect.

**Table 4:** Mean dietary intake or serum concentration of mediating nutrients by food insecurity level.

	Total	Full Security	Marginal/Low Security	Very Low Security	Daily Intake Req. or Ref. Range
Dietary Fiber (g)	**17.6 (±0.22)**	**18.0 (±0.24)**	**16.3 (±0.32)** ^1^	**16.2 (±0.60)** ^1^	28 [54]
Vitamin A (mcg)	**656 (±20)**	**689 (±24)**	**555 (±18)** ^1^	**551 (±20)** ^1^	900 [54]
Vitamin E (mg)	**9.20 (±0.12)**	**9.52 (±0.12)**	**8.20 (±0.20)** ^1^	**8.31 (±0.42)** ^1^	15 [54]
Vitamin C (mg)	**81.9 (±2.2)**	**83.8 (±2.2)**	**75.9 (±3.5)** ^1^	**75.8 (±6.3)**	90 [54]
Vitamin K (mcg)	126 (±4.5)	135 (±5.4)	**105 (±5.4)** ^1^	**89.1 (±5.6)** ^1^	120 [54]
Vitamin B1 (mg)	1.63 (±0.01)	1.65 (±0.02)	1.57 (±0.02) ^1^	1.65 (±0.06)	1.2 [54]
Vitamin B2 (mg)		2.21 (±0.02)	2.04 (±0.04) ^1^	2.12 (±0.09)	1.3 [54]
Potassium (mg)	**2730 (±25)**	**2790 (±26)**	**2520 (±40)** ^1^	**2560 (±99)** ^1^	4700 [54]
Zinc (mg)	11.3 (±0.09)	11.5 (±0.09)	11.1 (±0.22)	**10.8 (±0.35)**	11 [54]
Copper (mg)	1.30 (±0.02)	1.34 (±0.03)	1.17 (±0.02) ^1^	1.17 (±0.04) ^1^	0.90 [54]
Beta-carotene (mcg)	2380 (±100)	2600 (±118)	1740 (±120) ^1^	1600 (±150) ^1^	--
Serum Palmitoleic Acid (µmol/L)	264 (±4.8)	257 (±5.1)	277 (±8.1) ^1^	303 (±20) ^1^	110–113.0 [55]

Note. Bold indicates that average nutrient intake or serum concentration did not meet daily value or reference range. ^1^ Significant difference from full food security group (*p* < 0.05).

**Table 5:** Difference in intakes or serum concentrations of mediating nutrients between food-insecure, income-eligible FAP participants and non-participants.

	Received Benefits (*n* = 1410)	Never Received Benefits (*n* = 709)	*F*(1, 32)	*p*
Dietary Fiber	15.4 (±0.37)	17.8 (±0.70)	7.80	0.01
Vitamin A	544 (±21)	542 (±26)	0.004	0.95
Vitamin E	8.41 (±0.29)	8.45 (±0.46)	0.003	0.96
Vitamin C	80.6 (±6.4)	82.6 (±4.3)	0.063	0.80
Vitamin K	94.0 (±4.8)	99.2 (±6.2)	0.554	0.46
Vitamin B1	1.58 (±0.04)	1.61 (±0.04)	0.215	0.65
Vitamin B2	2.04 (±0.06)	2.00 (±0.06)	0.334	0.57
Potassium	2545 (±78)	2572 (±70)	0.065	0.80
Zinc	11.0 (±0.30)	11.2 (±0.34)	0.171	0.68
Copper	1.14 (±0.03)	1.19 (±0.04)	0.888	0.35
Beta-Carotene	1397 (±92)	1920 (±160)	8.80	0.01
Serum Palmitoleic Acid	313 (±18)	264 (±11)	6.08	0.02

## Data Availability

Publicly available datasets were analyzed in this study. The data can be found at https://cdc.gov/nchs/nhanes.

## Abbreviations

The following abbreviations are used in this manuscript:

FI	Food insecurity
FAP	Food assistance program
SNAP	Supplemental Nutrition Assistance Program
NHANES	National Health and Nutrition Examination Survey
SRI	Sleep regularity index

## Appendix A

### Appendix A2

**Table A1:** Relationships between nutrient intakes or serum concentrations and SRI score.

Nutrient	Mean	Parameter Estimate	*F* (1, 32)	*p*-Value	Literature Reference
Protein (g)	82.8 (±0.65)	1.70 (±0.52)	16.5	<0.001	Sutano et al. [28]
Carbohydrate (g)	259 (±2.3)	0.072 (±0.58)	2.21	0.15	Sutano et al. [28]
Total sugars (g)	114 (±1.2)	−0.376 (±0.45)	0.007	0.93	Alahmary et al. [49]
Dietary fiber (g)	17.7 (±0.29)	3.01 (±0.31)	97.2	<0.001	Ikonte et al. [30]
Total fat (g)	83.6 (±0.78)	1.14 (±0.41)	18.6	<0.001	Sutano et al. [28]
Cholesterol (mg)	290 (±3.5)	0.162 (±0.32)	1.21	0.28	Gangwisch et al. [50]
Vitamin A (mcg)	650 (±16)	1.36 (±0.26)	27.6	<0.001	Ikonte et al. [30]
Vitamin B1 (mg)	1.63 (±0.02)	1.36 (±0.39)	22.3	<0.001	Adventure-Heart et al. [51]
Vitamin B2 (mg)	2.18 (±0.03)	1.86 (±0.47)	28.9	<0.001	Adventure-Heart et al. [51]
Vitamin B3 (mg)	25.9 (±0.22)	1.07 (±0.44)	13.4	<0.001	Adventure-Heart et al. [51]
Vitamin B6 (mg)	2.17 (±0.03)	1.38 (±0.43)	20.5	<0.001	Adventure-Heart et al. [51]
Vitamin B12 (mcg)	5.05 (±0.09)	0.378 (±0.32)	4.23	0.27	Jahrami [31]
Vitamin C (mg)	82.4 (±1.9)	0.789 (±0.20)	15.9	<0.001	Ikonte et al. [30]
Vitamin D (mcg)	4.72 (±0.13)	0.305 (±0.21)	3.91	0.06	Ikonte et al. [30]
Vitamin E (mcg)	9.37 (±0.15)	0.434 (±0.14)	38.7	<0.001	Ikonte et al. [30]
Vitamin K (mcg)	123 (±3.9)	1.50 (±0.27)	29.4	<0.001	Ikonte et al. [30]
Beta-carotene (mcg)	2380 (±110)	1.02 (±0.15)	39.7	<0.001	Deng et al. [51]
Lycopene (mcg)	5200 (±120)	0.192 (±0.05)	21.8	<0.001	Deng et al. [52]
Total folate (mcg)	414 (±5.6)	2.13 (±0.39)	47.5	<0.001	An et al. [53]
Total choline (mg)	339 (±3.7)	0.162 (±0.32)	7.76	0.009	Ikonte et al. [30]
Vitamin B12 (mcg)	5.05 (±0.09)	0.378 (±0.32)	4.23	0.27	Jahrami [31]
Vitamin C (mg)	82.4 (±1.9)	0.789 (±0.20)	15.9	<0.001	Ikonte et al. [30]
Calcium (mg)	972 (±10)	1.74 (±0.42)	25.6	<0.001	Grandner et al. [68]
Phosphorus (mg)	1410 (±13)	1.91 (±0.59)	15.7	<0.001	Grandner et al. [69]
Magnesium (mg)	311 (±3.5)	3.70 (±0.45)	81.9	<0.001	Arab et al. [56]
Iron (mg)	15.1 (±0.16)	1.61 (±0.48)	23.8	<0.001	Ji et al. [32]
Zinc (mg)	11.3 (±0.10)	1.73 (±0.43)	32.3	<0.001	Ji et al. [32]
Copper (mg)	1.28 (±0.02)	3.32 (±0.47)	53.9	<0.001	Ji et al. [32]
Sodium (mg)	3560 (±29)	0.864 (±0.49)	8.33	0.007	Grandner et al. [68]
Potassium (mg)	2740 (±32)	2.89 (±0.58)	31.6	<0.001	Ji et al. [32]
Selenium (mcg)	266 (±6.4)	1.20 (±0.42)	11.5	0.002	Grandner et al. [68]
Caffeine (mg)	175 (±5.1)	0.441 (±0.09)	23.7	<0.001	Grandner et al. [69]
Theobromine (mg)	39.4 (±1.7)	0.350 (±0.07)	30.0	<0.001	Garbarino et al. [57]
Alcohol (gm)	11.5 (±0.54)	0.293 (±0.10)	14.3	<0.001	Park et al. [58]
Serum Vitamin B12 (pmol/L)	464 (±6.5)	0.769 (±0.65)	1.13	0.29	Beydoun et al. [33]
Serum Zinc (µmol/L)	12.7 (±0.09)	0.048 (±0.18)	1.04	0.32	Ji et al. [35]
Serum Palmitic acid (µmol/L)	2960 (±44)	−2.90 (±1.4)	8.39	0.007	Irmisch et al. [34]
Serum Palmitoleic acid (µmol/L)	266 (±6.4)	−2.82 (±0.71)	18.3	<0.001	Irmisch et al. [34]
Serum Oleic acid (µmol/L)	2250 (±36)	−3.15 (±1.2)	13.0	<0.001	Irmisch et al. [34]
Serum Linoleic acid (µmol/L)	3660 (±35)	4.26 (±1.9)	3.52	0.07	Irmisch et al. [34]
Serum Eicosadienoic acid (µmol/L)	23.4 (±0.33)	−0.427 (±1.2)	0.679	0.42	Irmisch et al. [34]
Serum Total Folate (nmol/L)	46.96 (±0.60)	3.24 (±0.51)	40.0	<0.001	An et al. [53]
Serum Folic acid (nmol/L)	2.289 (±0.14)	−0.082 (±0.34)	0.410	0.53	An et al. [53]

### Appendix A3

**Table A2:** Relationships between food insecurity and nutrient intake or serum concentrations.

Nutrient	Mean	*F* (2, 31)	*p*-Value
Protein (g)	82.8 (±0.65)	2.61	0.09
Carbohydrate (g)	259 (±2.3)	14.2	<0.001
Total sugars (g)	114 (±1.2)	27.6	<0.001
Dietary fiber (g)	17.7 (±0.29)	21.0	<0.001
Total fat (g)	83.6 (±0.78)	0.267	0.77
Cholesterol (mg)	290 (±3.5)	0.049	0.95
Vitamin A (mcg)	650 (±16)	15.6	<0.001
Vitamin A (mcg)	650 (±16)	15.6	<0.001
Vitamin B1 (mg)	1.63 (±0.02)	3.49	0.04
Vitamin B2 (mg)	2.18 (±0.03)	8.06	0.002
Vitamin B3 (mg)	25.9 (±0.22)	0.988	0.38
Vitamin B6 (mg)	2.17 (±0.03)	4.14	0.03
Vitamin B12 (mcg)	5.05 (±0.09)	2.92	0.07
Vitamin C (mg)	82.4 (±1.9)	6.03	0.006
Vitamin D (mcg)	4.72 (±0.13)	4.63	0.02
Vitamin E (mcg)	9.37 (±0.15)	34.4	<0.001
Vitamin K (mcg)	123 (±3.9)	16.6	<0.001
Beta-carotene (mcg)	2380 (±110)	22.1	<0.001
Lycopene (mcg)	5200 (±120)	1.57	0.22
Total folate (mcg)	414 (±5.6)	11.1	<0.001
Total choline (mg)	339 (±3.7)	3.53	0.04
Calcium (mg)	972 (±10)	1.16	0.33
Phosphorus (mg)	1410 (±13)	2.56	0.09
Magnesium (mg)	311 (±3.5)	34.2	0.09
Iron (mg)	15.1 (±0.16)	1.83	0.18
Zinc (mg)	11.3 (±0.10)	3.72	0.04
Copper (mg)	1.28 (±0.02)	21.5	<0.001
Sodium (mg)	3560 (±29)	1.31	0.29
Potassium (mg)	2740 (±32)	26.0	<0.001
Selenium (mcg)	266 (±6.4)	2.28	0.12
Caffeine (mg)	175 (±5.1)	2.08	0.14
Theobromine (mg)	39.4 (±1.7)	0.077	0.93
Alcohol (gm)	11.5 (±0.54)	0.255	0.78
Serum Vitamin B12 (pmol/L)	464 (±6.5)	3.04	0.06
Serum Zinc (µmol/L)	12.7 (±0.09)	1.41	0.26
Serum Palmitic acid (µmol/L)	2960 (±44)	1.18	0.32
Serum Palmitoleic acid (µmol/L)	266 (±6.4)	4.92	0.01
Serum Oleic acid (µmol/L)	2250 (±36)	0.493	0.62
Serum Linoleic acid (µmol/L)	3660 (±35)	0.887	0.42
Serum Eicosadienoic acid (µmol/L)	23.4 (±0.33)	3.42	0.05
Serum Total Folate (nmol/L)	46.96 (±0.60)	70.8	<0.001
Serum Folic acid (nmol/L)	2.289 (±0.14)	4.24	0.02
